# Males Have a Higher Energy Expenditure than Females during Squat Training

**DOI:** 10.3390/nu15153455

**Published:** 2023-08-04

**Authors:** Indya del-Cuerpo, Daniel Jerez-Mayorga, Luis Javier Chirosa-Ríos, María Dolores Morenas-Aguilar, Miguel Mariscal-Arcas, Alejandro López-Moro, Pedro Delgado-Floody

**Affiliations:** 1 Department of Physical Education and Sports, Faculty of Sports Sciences, University of Granada, 18071 Granada, Spain; delcuerpo@ugr.es (I.d.-C.); djerezmayorga@ugr.es (D.J.-M.); lchirosa@ugr.es (L.J.C.-R.); loles_bailen4@hotmail.com (M.D.M.-A.); 2 Strength & Conditioning Laboratory, CTS-642 Research Group, Department Physical Education and Sports, Faculty of Sports Sciences, University of Granada, 18071 Granada, Spain; 3 Exercise and Rehabilitation Sciences Institute, School of Physical Therapy, Faculty of Rehabilitation Sciences, Universidad Andres Bello, Santiago 7591538, Chile; 4 Department of Nutrition and Food Science, School of Pharmacy, University of Granada, 18071 Granada, Spain; mariscal@ugr.es (M.M.-A.); alexlopez@ugr.es (A.L.-M.); 5 Instituto de Investigación Biosanitaria de Granada (ibs. GRANADA), 18071 Granada, Spain; 6 Department of Physical Education, Sports and Recreation, Universidad de La Frontera, Temuco 4811230, Chile

**Keywords:** resistance training, energy cost, sex differences

## Abstract

The main objective of this study was to determine the differences in energy expenditure (EE) according to sex during and after two different squat training protocols in a group of healthy young adults. Twenty-nine Sports Sciences students volunteered to participate in this study. They attended the laboratory on four different days and completed four sessions: two sessions with 3 sets of 12 repetitions at 75% of their one-repetition maximum (RM) and two sessions with 3 sets of 30 repetitions at 50% of their 1RM. Energy expenditure was evaluated using an indirect calorimeter. Males consistently demonstrated higher EE in all sessions and intensities. The linear regression model identified a significant association between sex, BMI, and total EE across all sessions and intensities. In conclusion, males exhibited higher EE in both protocols (50% and 75% of 1RM) throughout all sessions. Furthermore, sex and BMI were found to influence EE in healthy young adults. Therefore, coaches should consider sex when assessing EE, as the metabolic response differs between males and females.

## 1. Introduction

Measuring energy expenditure (EE) during physical activity is extremely important not only in sports sciences but also in nutritional and clinical sciences [1]. Furthermore, it is imperative to maintain a high level of physical activity since it plays an essential role in the prevention and management of chronic conditions such as diabetes, obesity, cancer, cardiovascular, or cardiorespiratory diseases [2]. In addition, EE during physical activity is an important consideration in exercise prescription [3]. Literature has shown that physical activity is effective in increasing EE [4]. Specifically, strength training is not only useful for increasing muscle mass and strength, but among all its benefits, it also stands out for its effectiveness in increasing EE not only during training but also after exercise [5]. This is closely linked to the previously mentioned benefits, such as increased muscle mass and strength. Greater muscle mass and strength lead to an increase in basal metabolic rate [6]. As a result, there is an increase in EE, which helps reduce body fat and promotes overall health [7].

Various techniques can be used to calculate EE based on financial, human, and research objectives [8]. Questionnaires and activity diaries are generally seen as highly useful due to their low economic cost and ease of administration [9]. However, it is important to mention that their accuracy is reduced primarily because of the influence of recall bias [10]. On the other hand, doubly labeled water is the most accurate technique; however, effectively managing it poses challenges due to the need for highly specialized and costly equipment for data analysis [11]. As a result, employing this technique for extensive population studies is not feasible from a logistical standpoint [12]. Other techniques, such as calorimetry systems, have received increasing interest to quantify EE in the last decades, and they are more precise but much more expensive and not as accessible for most people [11,13]. Despite this, an abundance of different calorimetry systems have become available to researchers [14], including mobile [15] and computerized or fixed devices [16]. This growing interest in the use of indirect calorimetry can be seen in the increasing number of articles published in recent years that have employed these devices to evaluate EE during the performance of different types of physical exercise [16,17]. Finally, accelerometers are highly used because of their portability, versatility, and ease of use. Through its utilization, researchers can indirectly assess the intensity, frequency, and duration of individuals’ physical exercise [18]. However, compared to the aforementioned methods like indirect and direct calorimetry, accelerometers or activity trackers are considered less reliable and valid [19].

But EE is not equivalent for both sexes [20]. In adults, males tend to have a higher EE than females due to their higher muscle mass, which requires more energy for maintenance [21]. Regarding muscle strength, males tend to have higher strength levels due to the difference in muscle size, which favors males [22], which is larger in upper-body strength than it is in lower-body strength [23]. This suggests that sex plays a significant role in EE. Furthermore, these differences are not only found in terms of EE, but they also exist when it comes to strength training. According to the systematic review and meta-analysis conducted by Roberts, Nuckols, and Krieger [24], we know that both sexes adapt similarly to strength training for hypertrophy and lower-body strength, but there is a moderate difference in favor of females in the analysis of upper-body strength. This difference can be attributed to neural, muscular, and motor learning factors. Additionally, genetics, age, and lifestyle factors can also influence strength levels for both males and females [24]. 

During exercise, the energy supply is primarily determined by the intensity and duration of the exercise. For instance, anaerobic energy pathways have a higher power output in terms of ATP production rate but a smaller capacity for total ATP produced compared to aerobic pathways. Similarly, carbohydrate oxidation exhibits a higher power output in oxidative metabolism but a lower capacity than fat oxidation. This factor contributes to the decrease in power output when carbohydrate depletion occurs during long-duration, high-intensity exercise [25]. Additionally, during resistance exercise, the contributions of anaerobic energy systems play an important role in providing the energy for muscular contraction work and, therefore, are involved in energy expenditure during exercise [26].

The squat exercise is widely used in both conditioning and rehabilitation programs to build strength to activate and strengthen both the anterior and posterior chains [27]. In addition, this type of exercise is not only utilized in exercise programs [28], but it can also be used in everyday activities like going up and down the stairs, bending over to pick up shopping bags, and standing up from a seated position. Studies have shown that males and females employ different movements when performing a traditional back squat, potentially leading to the activation of different musculature during squat execution and impacting how the exercise is prescribed [29]. As a result of Mehls et al. [30], we know that males activate the biceps femoris muscle during the descending phase of the lift of the traditional back squat to a greater extent than females, but this activation is not statistically significant in the vastus lateralis, vastus medialis, rectus femoris, gluteus maximus, semitendinosus, and biceps femoris.

Despite this, as far as we know, there has been little work conducted evaluating EE during squatting and very little that evaluates EE during strength training in healthy young adults [31]. Most of the existing literature evaluates post-exercise EE in obese and elderly populations, and, above all, it is well studied during endurance training. Although squat exercise is widely used, the simultaneous evaluation of parameters such as strength, speed, power, work, and impulse using a single device [32,33] is a novel approach. This can be achieved by utilizing functional electromechanical dynamometry (FEMD), which is a valid and reliable isokinetic multi-joint device. Furthermore, the novelty of this study lies in the fact that, as far as we know, it is the first study to combine the use of FEMD with the assessment of EE. We hypothesized that males would have a higher EE during both squatting protocols. Therefore, the main objective of this study was to determine the differences in EE according to sex during and after two different squat training protocols in a group of healthy young adults.

## 2. Materials and Methods

### 2.1. Experimental Approach to the Problem

A repeated measures design was used to determine the differences in EE during two different protocols of acute squat exercise in FEMD according to sex. After the familiarization and repetition maximum (RM) determination session, each participant came to the laboratory four times over a two-week period, with at least 48 h in between visits. During each session, they performed 3 sets of 12 repetitions at 75% 1RM and 3 sets of 30 repetitions at 50% 1RM. The order of the protocols was randomized.

### 2.2. Subjects

Twenty-nine Sports Science students (age: 24.9 ± 4.6 years; height: 1.70 ± 0.1 m; body mass: 68.1 ± 12.9 kg; BMI: 23.5 ± 3.0 kg/m^2^), comprising thirteen males and sixteen females, volunteered to take part in this study (Table 1). Everyone who participated met the criteria for eligibility, which included (a) having no medical issues and (b) having at least one year of experience in muscle strength training immediately preceding the study. Before signing their consent forms, all participants were made aware of the specifics, purpose, and potential risks associated with the experiment. The study protocol was approved by the Committee on Human Research of the University of Granada (no. 2182/CEIH/2021) and was conducted in accordance with the Declaration of Helsinki.

During the initial contact with the participants to verify if they met all the inclusion criteria for participating in the study, females were asked various questions about their menstrual cycle. For example, they were asked about the start and end dates of their last menstruation, the duration of their menstrual cycle, whether they experienced severe pain or heavy bleeding, and if they used any hormone-based contraceptive methods. Based on the information provided by the female participants, we evaluated all of them during the luteal phase [34]. Furthermore, none of them used any hormone-based contraceptive methods, with only two experiencing severe pain and heavy bleeding.

### 2.3. Procedures

During the study, participants completed five sessions on separate days: one familiarization session and four experimental sessions. During these days, participants were asked to sleep at least 8 h, refrain from smoking, participate in strenuous exercise <12 h before testing, and eat 1 h before the session. They came to the laboratory at the same time each day (±1 h) and in similar environmental conditions (approximately 22 °C and 60% humidity).

It was decided to adjust the diet of all participants starting in the week prior to the study and throughout its duration to ensure that all participants started under the same nutritional conditions and that there were no external factors that could influence the results. This included eliminating all foods and beverages that could have an impact, such as caffeine and supplementation, among others. To achieve this, a Graduate in Human Nutrition and Dietetics was responsible for designing the same weekly diet for all participants during the week prior to the study and throughout the exercise period, tailored to their energy needs. To determine these energy needs, anthropometric measurements were taken for all participants one week before the study and during the subsequent weeks. These measurements included weight (using a professional TANITA SC-240-MA scale with a biological suite), height (using a portable Seca 213 Stadiometer), and skinfold measurements for the biceps, triceps, subscapular, abdominal, thigh, and mid-calf (using a Holtein HOL-98610ND mechanical caliper), as well as arm and mid-thigh circumferences (using a CESCORF measuring tape) by an ISAK level 1 anthropometrist. Basal EE was calculated using the Harris-Benedict formula [35], total EE was determined using the corresponding activity factor, and body fat percentage was estimated using the Foulkner formula [36]. Table 1 presents the sample menu provided to the participants.

**Figure 1:**
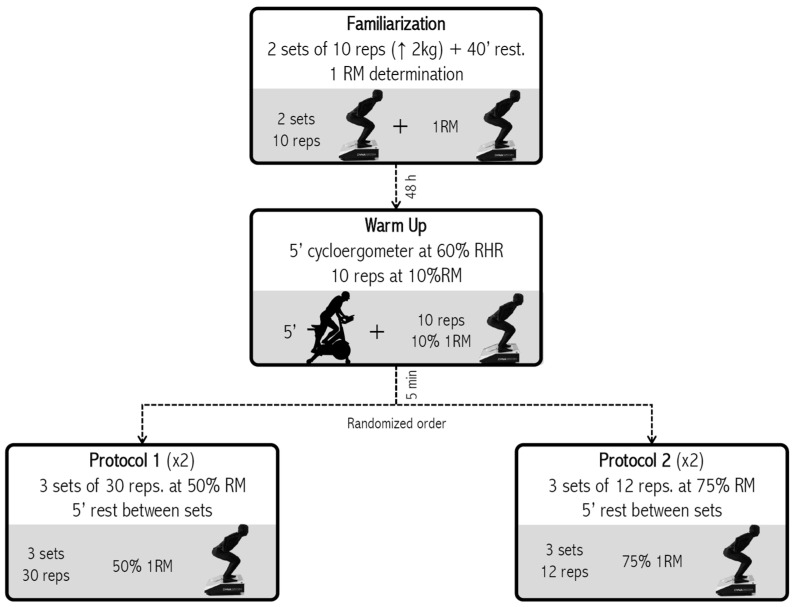
Protocol measurement of the squat exercise.

The individuals showed up for the study in accordance with the directions given by the researcher. When they arrived at the laboratory, as it was done in del-Cuerpo [32], they were fitted with the gas analyzer mask, and the gas analysis started. They remained seated in a relaxed position for 5 min. Once finished, they were equipped with a vest fitted with a carabiner attached to the FEMD cable. They then did a 5-min warm-up on a cycle ergometer at a rate of 60% of their reserve heart rate, followed by 10 repetitions of 10% of their 1RM to measure the angle of the exercise. Then they had 5 min of rest before performing 3 sets of 12 repetitions of 75% 1RM or 30 repetitions of 50% 1RM. Once they finished, they remained seated for 10 min for post-exercise gas analysis. Then, the indirect calorimeter and the vest were removed, and they were free to leave the laboratory. EE was measured indirectly using a metabolic cart, which analyzed the respiratory gases (usually expired gases) to determine the volume of air passing through the lungs, the amount of oxygen extracted from it (i.e., oxygen consumption or VO_2_), and the amount of carbon dioxide produced as a byproduct of metabolism, which is expelled into the atmosphere (CO_2_–VCO_2_). The order of the exercises was randomized, and they were given five minutes of rest between each set. The test-retest reliability of the FEMD for squat exercise was demonstrated in a previous study [32]. The protocol of the study is shown in Figure 1. 

On the initial visit to the laboratory, the participants had a 60-min session that included familiarizing themselves with the FEMD and determining their one-repetition maximum (1RM). This consisted of (a) a general warm-up of 2 sets of 10 squat repetitions with an initial load of 10 kg and increments of 2 kg on each repetition, with 40 s of rest between sets, and (b) directly estimating the participants’ squat 1RM by starting with a load of 100% of the body weight of a male and 80% of the body weight of a female, increasing in 4 kg increments up to 10 repetitions, as it was done in del-Cuerpo [32].

Once this is established, the participant will have several choices: (a) if the participant can perform more than one repetition, thus reaching failure. A rest of 5 min will occur, and then the initial load will be taken as the maximum load that was able to be achieved with further increments of 1 kg until the resistance becomes too strong (a maximum of five repetitions). The last repetition will be deemed the individual’s 1RM. (b) If they are not able to perform any repetitions, a rest of 2 min will occur, and then the initial load will be set at 90% of body weight for males and 70% of body weight for females with further increments of 1 kg until resistance is too strong (a maximum of five repetitions). The last repetition will be seen as the participant’s 1RM. (c) If the individual is only able to manage one repetition, a rest of 5 min will happen, and then the initial load will be the same as before with further increments of 1 kg until the resistance is too strong (a maximum of five repetitions). The last repetition will be viewed as the participant’s 1RM. Finally, (d) if the participant exceeds 120 kg (the load limit of the device), we will observe how many total repetitions they can do and estimate the 1RM with Lombardi’s equation [37]. 

The half squat was performed with FEMD (Dynasystem, Model Research, Granada, Spain), a validated isokinetic multi-joint device that allows us to evaluate the parameters of strength, speed, power, work, and impulse by using a single device [32,33]. The physical activity level of each participant was measured using the International Physical Activity Questionnaire (short form) (IPAQ), a suitable instrument for the assessment of physical activity in adults between 18 and 69 years of age [38].

The EE during both protocols was measured by using the FitMate™ metabolic system (Cosmed, Rome, Italy), a reliable and valid metabolic analyzer designed for measurement of oxygen consumption and EE during rest and exercise that measures breath-by-breath ventilation, expired oxygen, and carbon dioxide [35,36]. The indirect calorimeter does not require a warm-up time and performs self-calibration before testing each subject. After the warm-up, the mask was applied to the participant’s face and kept in place for ten minutes after completing the test. If the mask was not well adapted, a warning would be displayed on the device’s screen. All respiratory gas data was collected and analyzed from the beginning to the end of the protocol. The use of this device did not hinder the execution of the squat protocol in any way.

### 2.4. Statistical Analyses

The normal distribution of study variables was tested using the Kolmogorov-Smirnov test. For continuous variables, values are presented as the mean and standard deviation (SD). Differences according to sex in age, anthropometric variables (BMI), and physical activity according to the international physical activity questionnaire (i.e., low physical activity; MPA: moderate physical activity and vigorous physical activity) were determined using the t-test. A regression linear model was performed to determine the association among sex and BMI with total kcal in different intensities (i.e., 50% and 75%) adjusted by age. The results are presented as β (95% confidence intervals (CI)). All the statistical analyses were performed with SPSS statistical software version 23.0 (SPSS TM Inc., Chicago, IL, USA). The alpha level was set at *p* < 0.05 for statistical significance.

## 3. Results

Descriptive characteristics of the sample studied according to sex are shown in Table 2. There were no differences according to sex in body mass index or physical activity levels. Low physical activity, moderate physical activity, and vigorous physical activity. 

**Figure 2:**
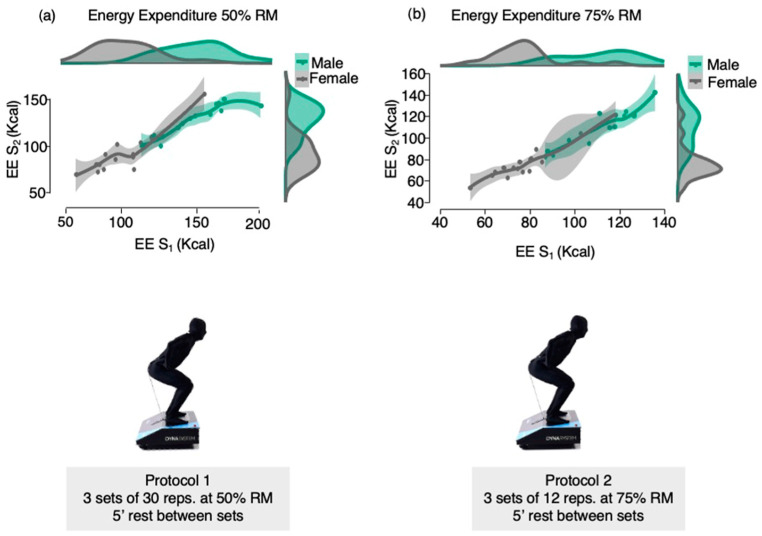
Total EE behavior during the two different squat protocols.

Male showed higher EE in all sessions and intensities (50% RM S1 male: 149.7 ± 21.9 vs. 50% RM S1 female: 103.4 ± 21.0, *p* < 0.001; 50% RM S2 male: 128.7 ± 16.8 vs. 50% RM S2 female: 93.7 ± 21.5, *p* < 0.001; 75% RM S1 male: 111.3 ± 16.3 vs. 75% RM S1 female: 78.0 ± 15.4, *p* < 0.001; 75% RM S2 male: 109.2 ±18.4 vs. 75% RM S2 female: 77.2 ± 16.4, *p* < 0.001; 50% RM S1 male: 31.1 ± 4.2 vs. 50% RM rest S1 female: 22.0 ± 4.9, *p* < 0.001; 50% RM rest S2 male: 16.4 ± 2.6 vs. 50% RM rest S2 female: 11.6 ± 2.3, *p* < 0.001; 75% RM rest S1 male: 17.3 ± 2.5 vs. 75% RM rest S1 female: 13.2 ± 3.3, *p* = 0.001; 75% RM S2 male: 16.5 ± 3.0 vs. 75% RM S2 female: 11.4 ± 2.1, *p* < 0.001) (Table 3). The Figure 2 show the total EE behavior during the two different squat protocols according to sex.

The linear regression model determined that there was a significant association between sex and BMI with total EE at 50% RM session 1 (sex, β: −39.49, *p* < 0.001, BMI, β: 0.77, *p* = 0.008, R^2^ = 0.66) and 50% session 2 (sex, β: −66.07, *p* < 0.001; BMI, β: 1.83, *p* = 0.001, R^2^ = 0.68) (Table 4). In relation to 75% season 1 (sex, β: −27.79, *p* < 0.001; BMI, β: 2.75, *p* = 0.008, R^2^ = 0.70) and 75% of season 2 (sex, β: −26.63, *p* < 0.001; BMI, β: 4.13, *p* = 0.001, R^2^ = 0.66), there was also a significant association between sex and BMI with EE (Table 5).

## 4. Discussion

To date, there has been little work conducted to determine whether females and males demonstrate differences in EE during resistance training. Thus, the objective of this study was to determine the differences in EE according to sex during and after two different squat training protocols in a group of healthy young adults. The main results of this study demonstrated that males had a higher EE in all sessions and intensities, and there was always a significant association between sex, BMI, and total EE.

Resistance training offers numerous benefits for those who engage in it, such as improved cardiovascular health due to an increase in heart rate and improved blood circulation [39]. It also promotes muscle growth, which improves body composition and reduces the risk of obesity and other lifestyle-related diseases [40]. All of this results in improved metabolic function and an increase in EE [41]. Muscle tissue is metabolically active, meaning it burns calories even when at rest, so as more muscle is built, the body burns more calories when it is inactive [42]. Additionally, these increases in metabolic function to replenish energy reserves and aid muscle building can lead to excess post-exercise oxygen consumption that can persist for up to 72 h after exercise [43], promoting an increase in EE even at rest and improved long-term metabolic efficiency [44]. Fortunately, resistance training and the subsequent increase in EE offer numerous benefits that aid in the prevention or control of chronic diseases such as obesity, diabetes, hypertension, heart disease, and certain types of cancer [45].

In this study, after conducting two sessions of 3 sets of 12 repetitions at 75% of 1RM and two more sessions of 3 sets of 30 repetitions at 50% of 1RM, as we supposed, it was demonstrated that males had a higher EE than females in both protocols and across all sessions. Our results are also consistent with those indicated by Lemmer et al. [46], who aimed to compare the effects of age and sex on strength training in relation to resting metabolic rate, EE during physical activity, and body composition. Lemmer et al. (2001) [46] were the first authors to suggest that the changes in EE in response to strength training were influenced by the sex of the subjects evaluated. After assessing EE before and after a 24-week strength training intervention, he observed a significant increase in EE in men, while this increase was not significant in females. More than 20 years ago, they proposed that this difference was primarily attributed to an increase in the metabolic activity of lean mass after strength training, which is linked to an increase in sympathetic nervous system activity [47].

From that moment until the present, both strength training and EE have been hot topics in society. However, despite this, the existing research on this matter is not so extensive. Nevertheless, the presented results continue to align with those published to date. Specifically, the presented findings correspond with those reported by Li et al. [20], who measured EE while walking on a treadmill with backpack and double pack loads and found that young, healthy female participants had a notably lower EE compared to male participants during a walking exercise. Now, with the passage of time, technologies have advanced, allowing for a deeper analysis of the obtained results, and the increase in EE in men can be related to other factors. As mentioned in another paragraph, it is now known that a higher percentage of muscle mass leads to an increase in EE [42]. This would suggest that, generally, males show a higher EE compared to females [21]. Furthermore, males also have a higher basal metabolic rate due to the presence of male hormones such as testosterone, which increase EE [48]. 

Regarding this, Trumble (2023) [49] observed that high levels of testosterone in males were positively associated with high EE while controlling for lean body mass and physical activity. This is because the anabolic effects of testosterone increase protein synthesis [50] and promote muscle cell proliferation through the activation of satellite cells [51], which require intensive calorie utilization and energy reserve diversion. Additionally, testosterone also enhances glucose uptake and utilization in muscle tissue [52]. Thus, we can explain why males who take testosterone supplements often exhibit a decrease in fat mass in addition to an increase in muscle mass [53]. On the contrary, Trumble (2023) [49] also observed that females have testosterone and lean muscle mass, but they do not exhibit the same associations between EE and testosterone. This difference could be attributed to the additional reproductive costs (gestation, lactation) that females bear, which males are not exposed to [54].

Focusing specifically on women, all of them were evaluated during the luteal phase of their menstrual cycle. This phase is commonly divided into three stages: (a) the early luteal phase, which starts after ovulation and involves the ruptured follicle transforming into the corpus luteum, which secretes progesterone and a small amount of estrogen; (b) the mid-luteal phase, which includes the peak of progesterone and a smaller second peak of estrogen, preparing the endometrium for the implantation of a fertilized egg; and (c) the late luteal phase, which ends with pregnancy if a fertilized egg is implanted. If the egg is not fertilized, the corpus luteum degrades, causing a decrease in progesterone and estrogen levels during the late luteal phase. Meanwhile, the cycle prepares to restart, and the uterine lining is shed, initiating menstruation once again [55,56,57]. During the menstrual cycle, the metabolic rate during sleep exhibits cyclical changes, with the highest point occurring during the late luteal phase. However, the increase in energy expenditure over a 24-h period is not statistically significant [34].

Additionally, the presented results indicate a significant correlation between sex, BMI, and total EE across all sessions and intensities. These findings are consistent with those of Pardo et al. (2014) [58], who studied the association of EE in conditions of extreme BMI in a group of 145 adult females, including 30 with anorexia, 66 with obesity, and 49 healthy normal-weight controls. They concluded that the obese subjects exhibited statistically lower daily physical activity with higher EE compared with the normal-weight subjects. So, now it is known that a higher BMI is accompanied by increased EE [59]. This is because those individuals with elevated BMI values, although they may have a greater amount of fat mass, also have a higher percentage of lean mass, which, as previously mentioned, leads to a high EE [58]. 

In addition, the present study found an inverse relationship between females and EE in all sessions and intensities. It has been previously reported that the male advantage in endurance performance is attributed to the sex difference observed in VO_2_max [60], which could explain the results. In fact, there are various physiological factors that could impact the variability of EE and interindividual responses. These factors include central factors involved in O_2_ delivery during exercise, such as diffusion across the pulmonary capillary membrane, pulmonary ventilation, cardiac output, and hemoglobin mass. Additionally, peripheral factors such as skeletal muscle blood flow and diffusion of O_2_ from the microcirculation into the muscle play a role. In females, the inability to match the high VO_2_max of their male counterparts is often attributed to these central factors, as they have smaller lungs, a smaller heart, and lower hemoglobin mass, which limits the capacity to deliver oxygen to the working muscles [60,61], affecting the EE. 

After learning about these new findings, it is interesting to note the practical applications that can be derived from them. Coaches can gain a better understanding of the differences in energy expenditure generated by the same training in males and females and apply this knowledge in practice. They should always consider that males generate a higher EE than females. Therefore, if a coach’s goal is to train a female to achieve a specific level of energy expenditure, they should prescribe a higher training volume compared to that prescribed for a male. Similarly, a similar situation occurs with BMI. A person with a higher BMI will generate a higher EE, which is important to consider when prescribing training.

Despite all this, the study has certain limitations that should be addressed in future research. The study included only young and healthy adults with a 1 RM of less than 160 kg. Therefore, future studies should include different populations, such as powerlifters, overweight or obese individuals, and people with other health conditions. Additionally, the study evaluated half squats, and it would be valuable to investigate the effects on EE with full squats and other different exercises and whether EE would change. Also, it would have been interesting to measure EE using an accelerometer as well, in order to compare the results obtained between the two different devices. Finally, it would have been highly interesting to conduct a study on sex hormones such as testosterone or estrogen in order to establish correlations with the obtained findings. All these limitations will be considered for future research.

## 5. Conclusions

In conclusion, the main findings of this study demonstrate that males had a higher EE in both protocols (50 and 75% 1RM) and during all sessions, as well as that EE depends on sex and BMI in healthy young adults. Despite this, the generation of new knowledge about the evaluation of EE in males and females can help us clarify the reason for these differences.

## Figures and Tables

**Table 1:** Sample menu provided to the participants.

	Monday	Tuesday	Wednesday	Thursday	Friday	Saturday	Sunday
Breakfast	1 glass of water; 1 tangerine; Glass of milk with pure cocoa (1 teaspoon of sugar) + 2 slices of toast with olive oil and crushed tomatoes	1 glass of water; 1 banana; Decaffeinated coffee with milk (1 teaspoon of sugar) + 2 slices of toast with olive oil and serrano ham (2 slices)	1 glass of water; 1 tangerine; Glass of milk with pure cocoa (1 teaspoon of sugar) + 2 slices of toast with avocado	1 glass of water; 1 pear; Decaffeinated coffee with milk (1 teaspoon of sugar) + 2 slices of toast with olive oil and crushed tomatoes	1 glass of water; 1 banana; Glass of milk with pure cocoa (1 teaspoon of sugar) + 2 slices of toast with olive oil and fresh cheese (2 slices)	1 glass of water; 1 pear; Decaffeinated coffee with milk (1 teaspoon of sugar) + 2 slices of toast with olive oil and serrano ham (2 slices)	1 glass of water; 1 banana; Glass of milk with pure cocoa (1 teaspoon of sugar) + 2 slices of toast with avocado
Snack	Avocado sandwich + 1 pear	Plain yogurt + 1 handful of walnuts	Rooibos tea (1 teaspoon of sugar) + olive oil and cooked ham sandwich (2 slices)	2 natural L-casei yogurts + 1 apple	Plain yogurt + 1 handful of almonds	Olive oil and sliced tomato sandwich + 1 tangerine	1 bowl of fruit salad with fresh orange juice
Lunch	1 glass of water; Medium-sized plate: lettuce and tomato salad; Large plate: roasted chicken thigh and potato; Bread (2 slices); Dessert: pomegranate	1 glass of water; Large plate: lentil stew with carrot and potatoes; Bread (2–3 slices); Dessert: 2 kiwis	1 glass of water; Large plate: peas with onion and ham + grilled hake with garlic; Bread (2–3 slices); Dessert: ½ cherimoya	1 glass of water; Large plate: stir-fried rice with beef and mushrooms; Bread (2 slices); Dessert: 1 pommegranate	1 glass of water; Large plate: lentil stew with carrot and potatoes; Bread (2–3 slices); Dessert: ½ cherimoya	1 glass of water; Medium-sized plate: lettuce and tomato salad; Medium-sized plate: pasta with tomato sauce and minced chicken meat; Dessert: 1 pommegranate	1 glass of water; Large plate: sautéed wild asparagus and shrimp scrambled with 2 eggs; Bread (2–3 slices); Dessert: plain yogurt
Snack	Plain yogurt + 1 handful of walnuts	Rooibos tea (1 teaspoon of sugar) + 1 pear	1 banana + handful of natural almonds	2 tangerines + handful of walnuts	Rooibos tea (1 teaspoon of sugar) + 1 pear	1 apple + 1 handful of natural almonds	Rooibos tea (1 teaspoon of sugar) + 1 toast with olive oil and turkey (1 slice)
Dinner	1 glass of water; Large plate: sautéed green beans with garlic + tuna omelette (2 eggs); Bread (2 slices); Dessert: ½ cherimoya	1 glass of water; Large plate: lettuce, pasta, fresh cheese, white asparagus, and tuna salad; Dessert: 1 pomegranate	1 glass of water; Large plate: zucchini and potato cream soup + skewer of chicken breast, cherry tomatoes, and peppers; Bread (2 slices); Dessert: plain yogurt	1 glass of water; Large plate: cauliflower scrambled with garlic and egg; Bread (2 slices); Dessert: 2 kiwis	1 glass of water; Large plate: zucchini and potato cream soup + skewer of salmon breast, cherry tomatoes, and peppers; Bread (2–3 slices); Dessert: 1 pomegranate	1 glass of water; Large plate: sautéed green beans with garlic + mushroom omelette (2 eggs); Bread (2 slices); Dessert: plain yogurt	1 glass of water; Large plate: lettuce, pasta, fresh cheese, white asparagus, and tuna salad; Dessert: 2 kiwis

**Table 2:** Descriptive characteristics of sample study according to sex.

	Total (n = 29)	Male (n = 13)	Female (n = 16)	*p*-Value	(*F*-Value)
	Mean ± SD	Mean ± SD	Mean ± SD		
Age (years)	24.9 ± 4.6	25.7 ± 3.9	24.3 ± 5.1	0.428	(0.649)
Anthropometric parameters					
BMI (kg/m^2^)	23.5 ± 3.0	24.6 ± 3.4	22.6 ± 2.4	0.076	(3.412)
Body composition					
Body fat mass (kg)	15.8 ± 5.3	14.7 ± 5.6	16.7 ± 5.2	0.345	(0.923)
Fat-free mass (kg)	52.4 ± 11.4	63.0 ± 7.2	43.9 ± 5.1	<0.001	(1.160)
Physical activity (IPAQ)					
LPA (days)	4.7 ± 2.9	4.9 ± 2.9	4.6 ± 2.9	0.840	(0.042)
LPA (min/day)	37.9 ± 36.5	36.2 ± 32.5	39.4 ± 40.4	0.818	(0.054)
MPA (days)	2.5 ± 2.3	3.2 ± 2.6	2.0 ± 2.0	0.189	(1.820)
MPA (min/day)	66.0 ± 78.5	77.7 ± 96.0	56.6 ± 62.7	0.481	(0.510)
VPA (days)	3.7 ± 1.6	3.8 ± 1.4	3.6 ± 1.7	0.810	(0.059)
VPA (min/day)	90.5 ± 37.6	92.3 ± 31.7	89.1 ± 42.8	0.822	(0.052)

BMI: body mass index; SD: standard deviation; IPAQ: international physical activity questionnaire; LPA: low physical activity; MPA: moderate physical activity; VPA: vigorous physical activity. Data is shown as mean and (SD). Comparisons were measured by a general linear model using a *t*-test. *p* < 0.05.

**Table 3:** Comparison of EE according to intensity.

	Total (n = 29)	Male (n = 13)	Female (n = 16)	*p*-Value (*F* Value)
	Mean ± SD	Mean ± SD	Mean	
Total EE for 50% RM S1 (kcal)	124.1 ± 31.4	149.7 ± 21.9	103.4 ± 21.0	*p* < 0.001 (33.510)
Total EE for 50% RM S2 (kcal)	109.4 ± 26.1	128.7 ± 16.8	93.7 ± 21.5	*p* < 0.001 (22.964)
Total EE for 75% RM S1 (kcal)	92.9 ± 22.9	111.3 ± 16.3	78.0 ± 15.4	*p* < 0.001 (31.964)
Total EE for 75% RM S2 (kcal)	91.6 ± 23.5	109.2 ± 18.4	77.2 ± 16.4	*p* < 0.001 (24.614)
EE for 50% RM 10 min rest S1 (kcal)	26.1 ± 6.5	31.1 ± 4.2	22.0 ± 4.9	*p* < 0.001 (28.009)
EE for 50% RM 10 min rest S2 (kcal)	13.7 ± 3.4	16.4 ± 2.6	11.6 ± 2.3	*p* < 0.001 (27.610)
EE for 75% RM 10 min rest S1 (kcal)	15.0 ± 3.5	17.3 ± 2.5	13.2 ± 3.3	*p* = 0.001 (13.667)
EE for 75% RM 10 min rest S2 (kcal)	13.7 ± 3.6	16.5 ± 3.0	11.4 ± 2.1	*p* < 0.001 (28.724)

SD: standard deviation; RM: repetition maximum; S1: session 1; S2: session 2. Data is shown as mean and (SD). Baseline comparisons were measured by a general linear model, using a *t*-test. *p* < 0.05.

**Table 4:** Association between total EE 50% intensity (kcal), sex, and BMI.

	50% RM kcal Total S1				50% RM kcal Total S2			
	β (95%CI)	Beta	SE	*p*-Value	β (95%CI)	Beta	SE	*p*-Value
Sex	−37.28 (−52.26; −22.31)	−0.60	7.27	*p* < 0.001	−26.63 (−39.95; −13.32)	−0.52	6.46	*p* < 0.001
BMI (kg/m^2^)	4.19 (1.67; 6.72)	0.40	1.23	0.002	4.13 (1.88; 6.38)	0.47	1.09	*p* = 0.001

RM: repetition maximum; S1: session 1; S2: session 2; CI: 95% confidence interval; SE: standard error; BMI: body mass index. Data is shown as β (95% CI). Male as reference value in sex.

**Table 5:** Association between total EE 75% intensity (kcal), sex, and BMI.

	75%RM kcal Total S1				75% RM kcal Total S2			
	β (95% CI)	Beta	SE	*p*-Value	β (95% CI)	Beta	SE	*p*-Value
Sex	−27.79 (−39.49; −16.10)	−0.61	5.68	*p* < 0.001	−24.52 (−36.07; −12.96)	−0.53	5.61	*p* < 0.001
BMI (kg/m^2^)	2.75 (0.77; 4.72)	0.36	0.96	*p* = 0.008	3.78 (1.83; 5.73)	0.48	0.95	*p* = 0.001

RM: repetition maximum; S1: session 1; S2: session 2; CI: 95% confidence interval; SE: standard error; BMI: body mass index. Data is shown as β (95% CI). Male as reference value in sex.

## Data Availability

The datasets generated for this study are available on request to the corresponding author.
